# Cerium oxide nanoparticles modulating the Parkinson's disease conditions: From the alpha synuclein structural point of view and antioxidant properties of cerium oxide nanoparticles

**DOI:** 10.1016/j.heliyon.2023.e21789

**Published:** 2023-10-31

**Authors:** Xiaomei Yao, Yichao Guan, Jianli Wang, Dong Wang

**Affiliations:** a Department of Geriatrics, Central Hospital Affiliated to Shandong First Medical University, No. 105, Jiefang Road, Jinan City, Shandong Province, 250013, China; b Department of Neurology, Central Hospital Affiliated to Shandong First Medical University, No. 105, Jiefang Road, Jinan City, Shandong Province, 250013, China

**Keywords:** Parkinson's disease, Cerium oxide, Nanoparticles, Alpha synuclein

## Abstract

Parkinson's and Alzheimer's disease is the main cause of dementia, which is associated with the progressive deterioration of the intelligence and senses. Free radicals are created during oxidative stress in cells, which are considered one of the destructive factors in neurodegenerative diseases. In this study, the antifibrillar and antioxidant properties of cerium oxide nanoparticles (CeO_2_ NPs) were investigated experimentally and theoretically. The CeO_2_ NPs were synthesized and analyzed to reveal the physicochemical and biological properties. The results showed that the CeO_2_ NPs have unique properties with potent antioxidant activities. The experimental and computational studies showed that the CeO_2_ NPs interact with the active site of Alpha-synuclein. The existence of hydrogen bonding between O atoms of CeO_2_ NPs and N–H of adjacent acid amines and the equilibrium distances were confirmed by 1.751 (Leu100), 1.786 (Gln99) and 2.213 Å (Lys97). The minimum free energy binding of L-DOPA drug (as positive control) and CeO_2_ NPs were negative, resulting interaction between compounds and protein. As a result, these compounds inhibited Alpha-synuclein protein aggregation. In addition, that CeO_2_ NPs strongly binds with receptor by relative binding energy as compared with L-DOPA drug. These findings revealed that CeO_2_ NPs prevent Alpha-synuclein fibrillation and can be applied as nano-drug against the Parkinson's disease.

## 1 Introduction

Parkinson's disease (PD) is the most prevalent and progressive neurodegenerative disorder (ND) recorded, and its incidence is rising in elderly populations. PD is estimated to affect around 3 % of individuals over the age of 65 [1,2]. From a clinical point of view, this disease is classified as motor deficits, but these motor deficits are usually associated with a decrease in the level of consciousness and cognitive abilities in patients [3,4]. The clinical symptoms of this disease include tremors while resting and slowness of movements [5]. If the disease progresses, other symptoms such as lack of movement and instability may be seen in patients [6]. This results in a subsequent loss of dopamine in the striatum, which is an important neuropathological signature [7]. In addition, these complications are the result of the accumulation of a group of protein substances inside neurons known as Lewy bodies [8]. The major part of Lewy bodies consists of amyloid fibrils of alpha-synuclein protein [9]. It was discovered that the fibrils of Lewy bodies and the neurites of Lewy bodies both contained a significant amount of α-synuclein. Despite the fact that in most cases the occurrence of this disease is non-hereditary or isolated, studies have indicated that about 5–10 % of these cases have a genetic basis and fall under the familial type of Parkinsonism [10,11]. In addition, rare familial variants of PD6 have been linked to missense mutations in the α-synuclein genes, such as A53T, A30P, and E46K. In addition, gene polymorphisms have been categorized as risk factors for either idiopathic or sporadic Parkinson's disease [[12], [13], [14], [15], [16]].

In the current environment, attempts have been undertaken the invention of pharmacological methods against PD. Some examples of these pharmacological approaches include medications that can either expedite the amount of dopamine in the central nervous system or the stimulation of dopamine receptors [17]. However, the use of these medicines is restricted to the DAergic targeting therapy method, and they only served to alleviate the symptoms; there is little to no evidence to suggest that such treatments helped to slow down the degeneration of DA neurons [[18], [19], [20]]. In addition, it has been observed that medication therapies such as L-DOPA might generate a number of negative side effects. In addition, neuroprotection clinical trials have not yet unequivocally identified a medication that can either slow down or stop the progression of the disease [21,22].

Creating an effective treatment method have the ability to penetrate the blood brain barrier (also known as the BBB) is one of the primary issues associated with neurological illnesses. Due to proprietary transmission and the ability to penetrate biological barriers, nanoparticles have emerged as a potentially game-changing treatment for neurodegenerative illnesses such as PD and strokes. However, recent research has shown that certain types of nanomaterials have inherent therapeutic potential and can actively regulate cell function by controlling cellular processes [23]. These nanomaterials have been proven to have anti-angiogenic and antioxidant capabilities. It has been demonstrated that graphene and superparamagnetic iron-oxide nanoparticles (SPIONs), in addition to other nanoparticles (NPs), can prevent the fibrillation of amyloid beta during NDs. In particular, nanoparticles composed of cerium oxide (CeO_2_) have been shown to have neuroprotective action due to the antioxidant and anti-apoptotic characteristics that they possess [[24], [25], [26], [27]].

Alpha-synuclein protein is abundantly expressed in the central nervous system and is part of a small family of soluble proteins known as the synuclein family and has four members such as alpha-synuclein, beta-synuclein, gamma-synuclein and synoretin [28]. The sequence of this family of proteins shows high conservation between different animal species [29]. The exact function of alpha-synuclein remains unknown, but it appears to be involved in processes such as neuronal differentiation, axonal transport, and synaptic vesicle trafficking [30,31]. Some evidence has indicated that the accumulation of alpha-synuclein proteins in the brain of patients with Parkinson's disease is the primary factor in triggering the reactions that eventually lead to the death of nerve cells [32]. Therefore, knowing the structural details of alpha-synuclein fibrils at the molecular level is the first step in the way to find suitable treatment methods for this disease [33].

Nanotechnology and nanomedicine have the possibility to advance medical treatment by constructing substances with improved biological effects at the atomic scale. This might be accomplished through the application of nanotechnology and nanomedicine. Through the development of nanopharmaceuticals, it is possible that we may be able to create medications with heightened and more targeted effects, thereby delivering greater treatment for the disease [34,35]. The purpose of this research is to investigate the role that cerium oxide nanoparticles, a powerful nanoparticle antioxidant, play in the maintenance of neuronal function in a preclinical model of Parkinson's disease.

## 2 Chemicals and experiments

### 2.1 Chemicals

Solvents (methanol, hexane, dichloromethane, ethyl acetate, and normal butanol), salts and all the compounds were purchased from Merck Company. Alpha-synuclein protein monomers, Thioflavin T (ThT), Congo red, and all the compounds (DPPH, MTT, DMSO, DMEM, FBS, antibiotics (Streptomycin/Penicillin) and so on) for conducting biological tests were purchased from Sigma and GIBCO, UAA.

### 2.2 Experimental details

The hydroxide-mediated strategy was used to carry out the synthesis process. This method is superior to others in that it allows for the achievement of a uniform distribution of particle size [[36], [37], [38]]. Both solutions (Ce(NO3)3•6H2O (0.1 M) and NaOH (0.3 M)) were made by diluting the respective compounds with 200 mL of deionized water each and placing the mixtures in two separate beakers of the same volume (250 mL). The burette stand was outfitted with a freshly cleaned burette. A magnetic stirrer was positioned underneath the burette, and a beaker containing a 0.1 M solution of cerium nitrate was positioned on top of the magnetic stirrer in order to ensure that the solution was being stirred continuously. Following this step, the cerium nitrate solution was placed below the burette, and the 0.3 M solution of NaOH was withdrawn from the burette and dripped into it. At the conclusion of the procedure, a solution containing precipitate was obtained. This solution was centrifuged for 15 min at 8000 revolutions per minute in order to force the precipitate to fall to the bottom of the container. The remaining supernatant was thrown away and the precipitate has washed a total of four times with water, once each with ethanol and isopropanol, and finally with water again. Following the completion of this procedure, the resulting precipitate was collected on a glass plate and then baked at a temperature of 200° Celsius in a hot air vacuum oven. After all of this had been completed, a mortar and pestle were used to break up the dried precipitate into smaller and smaller pieces.

### 2.3 Characterizations of CeO_2_ NPs

Tabletop Hendon G2 Pro Phenom SEM, manufactured by FEI Company and located in Hillsboro, Oregon, USA, was the instrument of choice for the scanning electron microscopy examination. The scanning electron microscope (SEM) is a versatile piece of equipment that may offer qualitative information about the material including information regarding its shape, topography, content, and crystallographic structure. In order to get the sample ready for the SEM, a piece of double-sided carbon adhesive tape was cut to the desired form, and then one side of the tape with exposed adhesive was affixed to the specimen holder. The other side of the tape has a thin coating of liner on top of it. A trace amount of cerium oxide nanoparticles was dissolved in ethanol, and then the mixture was sonicated to ensure that the nanoparticles were evenly dispersed throughout the ethanol. As soon as the sonication was complete, the top layer of the liner of the tape was peeled off, and then 10 L of the sonicated solution was pipetted onto the adhesive portion of the specimen holder. After being dried, the sample was then placed into the apparatus so that the SEM examination could begin.

The TEM analysis was conducted using a Hitachi H-7600 tungsten-tip apparatus, operating at an accelerating voltage of 100 kV. The nanoparticles were subjected to examination following their dispersion in water and subsequent deposition onto TEM grids and were coated with gold.

The UV–Visible–NIR (PerkinElmer, Lambda 35) instrument was utilized to assess the optical characteristics of the samples. The particle size distribution was assessed using the Zetasizer Nano Series analyzer (Zetasizer Nano ZS90, Malvern). The X-ray powder diffraction (XRD) patterns were obtained using a Bruker D8 diffractometer equipped with a scintillation counter, operating in reflection mode with CuKα radiation. The examination of crystallite domain sizes (D) was conducted using X-ray diffraction (XRD) peaks, employing Scherrer's equation: D = 0.9λ/(βcosθ), where λ represents the wavelength of X-ray (λ = 0.15418 nm), θ is the Bragg's diffraction angle, and β is the real half peak width of the X-ray diffraction lines. The nanoparticles were analyzed using Fourier Transform Infrared Spectroscopy (FTIR) with the Nicolet Nexus 670 instrument (Nicolet instruments, Madison, WI, USA). The instrument was operated in absorbance mode within the spectral range of 650 cm-1 to 4000 cm-1, and numerous scans were performed for accurate analysis.

### 2.4 Antioxidant activity

The methodology outlined by Refs. [39,40] was followed in order to evaluate the phytofabricated CeO_2_ NPs for their ability to scavenge free radicals. In order to determine the antioxidant capacity of CeO_2_ NPs at varying concentrations (10, 20, 40, 100, and 200 g/ml), experiments were conducted. The combination of ingredients for the reaction comprised one mL of 2, 2-diphenyl-1-picrylhydrazyl (DPPH) and 1 mL of CeO_2_ NPs in varying concentrations. After vigorously shaking the mixture and incubating it at 25° Celsius for 30 min, the absorbance was determined using a wavelength of 517 nm. All of the chemicals were found in the control sample, with the exception of CeO_2_ NPs. Ascorbic acid was utilized as the standard reagent in this experiment.

### 2.5 Induction of fibrillation in alpha-synuclein protein

Alpha-synuclein is one of the proteins which is monomer in normal state and without regular secondary structure, but in disease conditions it turns into fibrils with regular structure of beta sheets, which can be induced under specific conditions in the laboratory. To induce fibrillation, 0.5 mg/ml of alpha-synuclein protein was heated in 25 mM Tris buffer (pH = 7.2) at 37° Celsius, so that the resulting solution was heated using a small magnet with a constant speed [41].

### 2.6 Thioflavin-T (ThT) assay

**Scheme 1:**
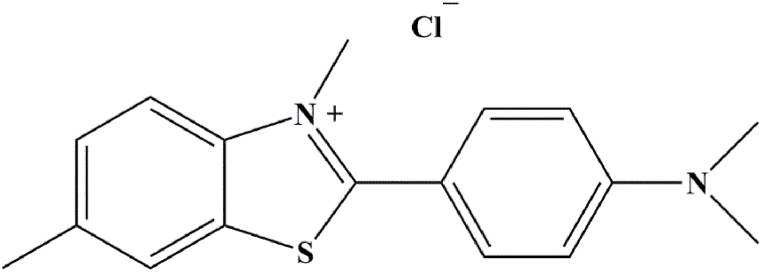
Representation of 4-(3, 6-dimethyl-1, 3-benzothiazol-3-ium-2-yl)-N, N-dimethylaniline; chloride (Thioflavin-T) molecular structure. It is a chemical compound with the chemical formula C_17_H_19_ClN_2_S and molar mass of 318.86 g/mol. ThT is a cell-permeable benzothiazole dye that exhibits increased fluorescence when bound to amyloid fibrils.

In order to investigate the process of fibrillation of alpha-synuclein and the inhibitory effect of nanoparticles on the protein fibrillation process, the fluorescence emission of ThT has been used. ThT is a cell-permeable benzothiazole dye that exhibits increased fluorescence when bound to amyloid fibrils (Scheme 1). For this purpose, 880 μL of ThT solution diluted with 20 μL of protein sample was heated for 5 min in the dark and at room temperature. By exciting the sample at a 440 nm of wavelength, the fluorescence emission was recorded in the range of 450–550 nm [42].

### 2.7 Atomic force microscopy (AFM)

Atomic force microscope imaging technique was used to examine the structure and morphology of amyloid conditions and amyloid fibril formation. In this regards, 20 μL of the samples (0.5 mg/ml α-synuclein incubated in the presence and absence of cerium oxide nanoparticles (0.5 mg/ml), 37 °C, and pH 7.4) was placed on a fresh and smooth layer of mica washed with ethanol. After about 10 min, the mica was washed with glycine buffer and then dried under nitrogen gas. The samples were selected according to the ThT fluorescence kinetic curve. In this investigation, the tapping mode method and the Dual Scope probe Scanner (DSS) atomic force microscopy model was used.

### 2.8 Circular dichroism (CD) spectroscopy

Circular Dichroism (CD) is the different absorption of two left-handed and right-handed circularly polarized light by an optically active sample. Almost all molecules synthesized by living organisms are optically active. Far- Ultraviolet (UV) region between the wavelengths of 190–260 nm allow us to quantitatively investigate the content of the secondary structure of a protein. In this region of the spectrum, the main absorbing chromophore is the peptide bond. In this study, the circular dichroism spectra of the protein were obtained using an AVIV 215 polarimeter spectrometer. A concentration of 0.5 mg/ml of α-synuclein and cuvette with a thickness of 0.1 cm were used to measure the circular dichroism spectra in the far ultraviolet range. The results obtained from two periodic colorimetry were analyzed using CDNN software and the percentage of each of the second structures of α-synuclein was calculated at different conditions (incubated in the presence and absence of cerium oxide nanoparticles (0.5 mg/ml), 37 °C, and pH 7.4).

### 2.9 Congo red light absorption

**Scheme 2:**
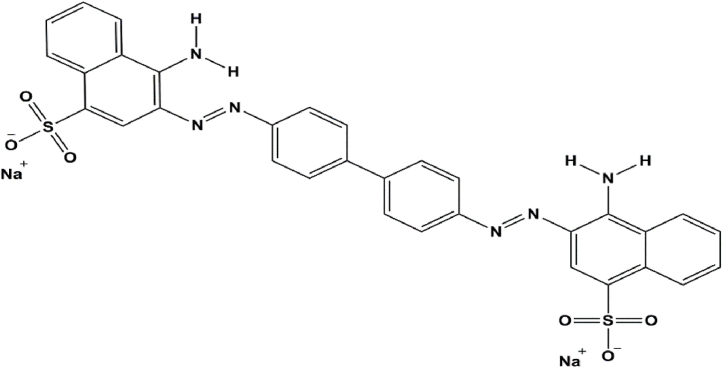
Representation of Congo red molecular structure (3, 3’-(biphenyl-4, 4′-diyldidiazene-2, 1-diyl) bis (4-aminonaphthalene-1-sulfonate)). It is a sodium salt of benzidine-diazo-bis-1-naphthylamine-4-sulfonic acid. It is used as biological ink and acid-base indicator, which is red in alkaline solutions and blue in acidic solutions.

To evaluate the amyloid fibrils formation, the Congo red (Scheme 2) colorimetric test can also be used. Congo red has the ability to bind to beta sheets and is orange in its natural state, but when fibrils are added to it, it changes color to purple and a shift to longer wavelengths is observed in its absorption spectrum. Therefore, it was used to investigate the process of protein fibrillation. The optical absorption of the samples was recorded at a distance of 400–600 nm. For this purpose, 40 μL of heated samples were added to 960 μL of fresh Congo red solution (10 μM) in 15 mM sodium phosphate buffer and the light absorption spectrum was read in the wave length of 400–600 nm. The increase in light absorption of Congo red and the transmission of its emission spectrum towards the red wavelength indicates the presence of amyloid structures [42].

### 2.10 Model construction and computational details

**Fig. 1:**
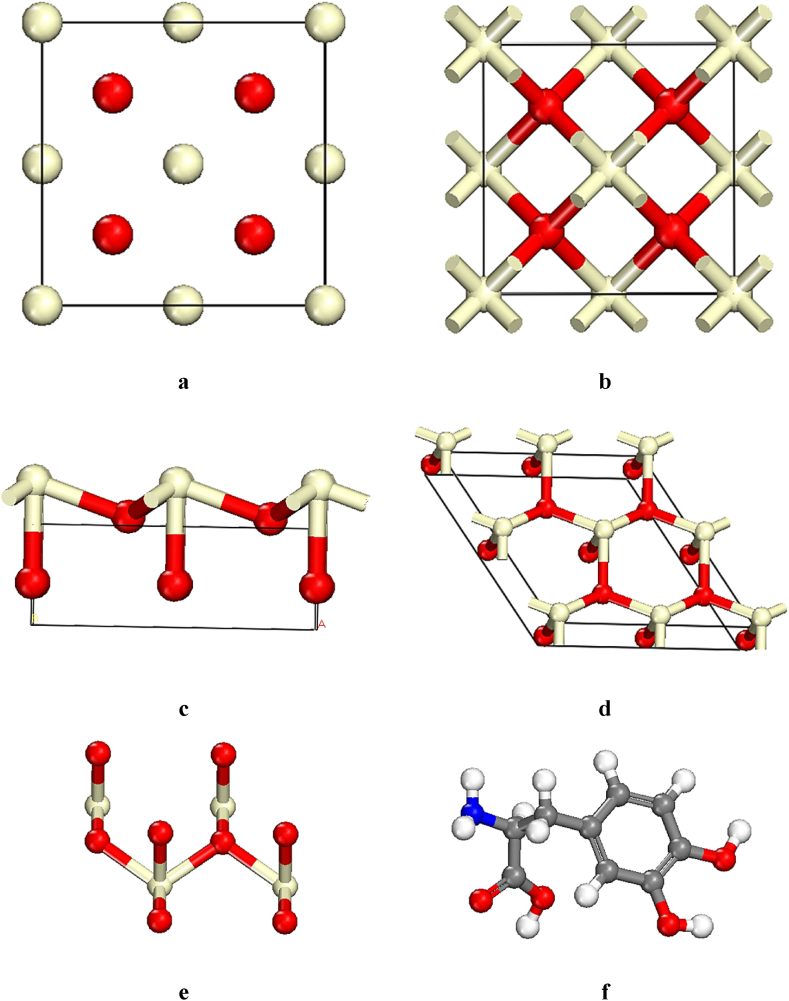
Snapshots of the side view (a and b); CeO₂ with code; mp-20194, Point Group m $\bar{3}$ m, (c and d); CeO₂ constructed based on (111) surface and thickness of 1.00 Å, (e); CeO₂ without cell and (f); Levodopa. Colored balls are oxygen (red), orange (Si), blue (N), light yellow (Ce), and white (H). (For interpretation of the references to color in this figure legend, the reader is referred to the Web version of this article.)

All compounds containing of Levodopa and CeO_2_ were optimized based on dispersion-corrected density functional theory (DFT-D) using generalized gradient approximation (GGA) and Perdew–Burke–Ernzerhof (PBE) hybrid functional by DMol3 in Material Studio 2017. Spin unrestricted and Multipolar expansion: Quadrupole were selected for this research [43,44]. CeO_2_ as cubic (code; mp-20194, Point Group m $\bar{3}$ m) with a = b = c = 5.47, 5.590 Å, α = β = γ = 90.000° and slab position along the c-axis; 1 Å was downloaded from https://materialsproject.org/(Fig. 1a–b) [45]. The unit structure of new CeO_2_ containing of different dimensions a = 7.732, b = 7.732, c = 2.367 Å, α = β = γ = 90.000° was constructed based on (111) surface and thickness of 1.00 Å (Fig. 1c–d). CeO_2_ without cell unit and Levodopa were prepared to optimize as showed in Fig. 1e–f.

### 2.11 Molecular docking method

In order to predict the *α*-synuclein activity in presence of CeO_2_ and L-DOPA drug molecule, the *α*-synuclein (1XQ8) and CeO_2_ were prepared and used as PDB and Mol2 files. *α*-synuclein was downloaded from https://www.rcsb.org/structure/1xq8. At the first time, cavities were selected to interact compound and receptor. Then, intemal ES, Intemal HB and Sp2-Sp2 Torsion were selected and Energy Minimization was calculated. The grid box volume was adjusted to 228.31 × 17.75 × -12.77 Å in the X, Y, and Z axes, respectively. To obtain output, Mol2 was saved. At last, the output was used to evaluate interactions of acid amines with compounds, and for this aim, Molegro Virtual Viewer, Discovery Studio Client and Chimera were used [46,47].

### 2.12 Statistical analysis

The results were presented as an average along with the standard deviation. The data was analyzed with SPSS software, and the significance of the results between the two groups was measured by the student-test method. At least P < 0.5 is considered significant.

## 3 Results and discussion

### 3.1 Properties of CeO_2_ NPs

**Fig. 2:**
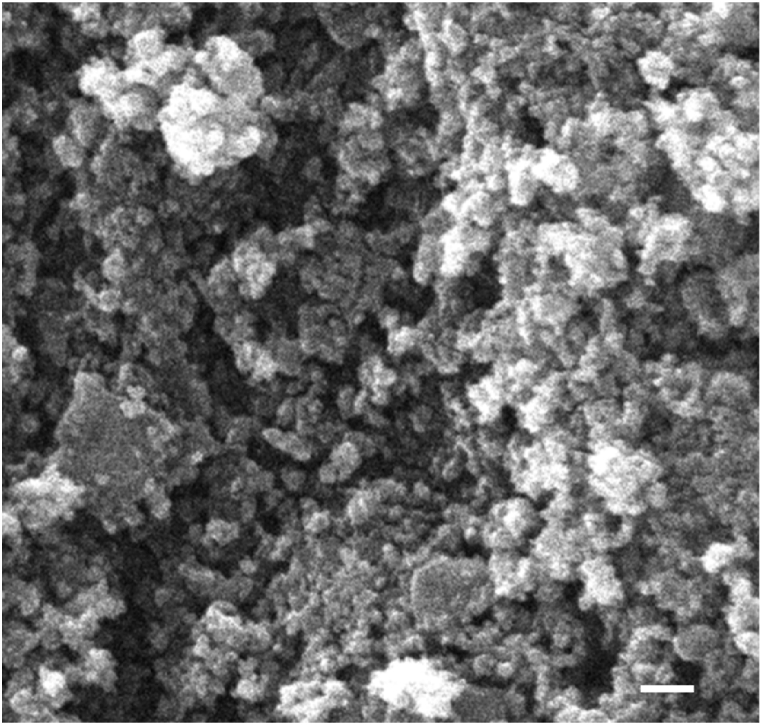
SEM image representing the particle distribution of CeO_2_NPs.

The CeO_2_ NPs were effectively synthesized by employing the hydroxide-mediated strategy, and the resulting NPs possessed a yellowish-white coloration. This is one of the straightforward and cost-effective approaches of synthesizing the CeO_2_ NPs. One of the important precursors that are employed in a variety of different synthesis techniques is cerium nitrate hexahydrate. In the hydroxide-mediated technique, cerium nitrate hexahydrate is first dissolved in DI water to produce a homogenous solution. This solution is then used to facilitate the transition from the cerium state Ce3+ to the cerium state Ce4+, which is followed by a reaction between NaOH and cerium cerium nitrate hexahydrate. After putting the CeO_2_ NPs through a scanning electron microscopy (SEM) examination, the researchers got a picture that showed a cluster of particles that were evenly dispersed over the whole thing, as can be seen in Fig. 2. When the NPs were measured with the program image J, the results showed that the average size of the particles collected was lower than 100 nm, indicating the presence of NPs.

**Fig. 3:**
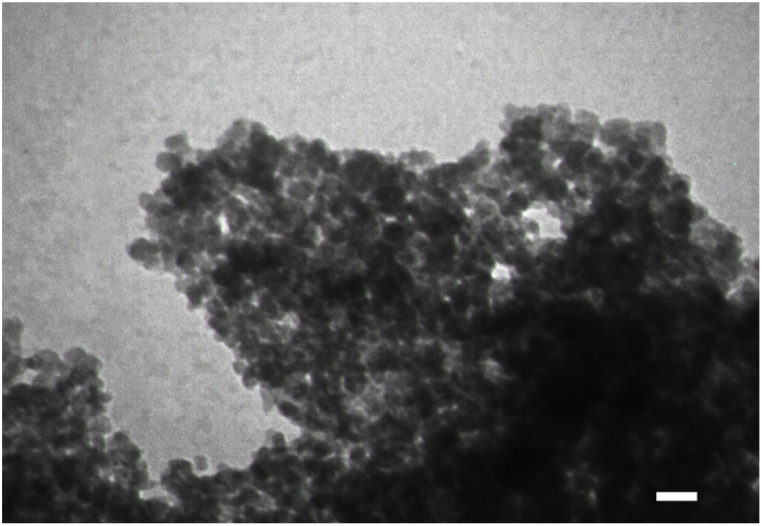
TEM image representing the particle distribution of CeO_2_ NPs.

TEM imaging was also used to visualize the synthesized CeO_2_ NPs and the results are presented in Fig. 3. (Methodology for TEM) The results showed that the synthesized CeO_2_ NPs have a uniform morphology and are monodispersed.

### 3.2 CeO_2_ NPs characterizations

**Fig. 4:**
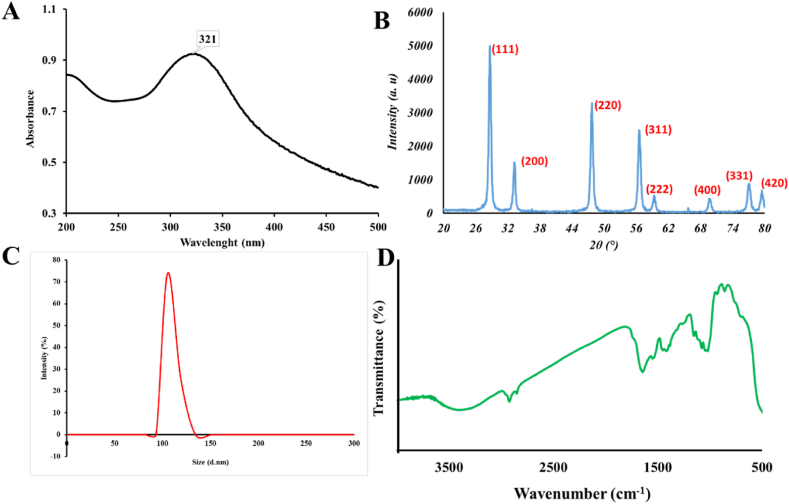
Characterization results of the synthesized CeO_2_ NPs. (A) UV–Vis absorbance spectra, (B) XRD pattern, (C) DLS diagram, and (D) FTIR spectra of the synthesized CeO_2_ NPs.

The optical absorption spectra of the synthesized CeO_2_ NPs in UV–visible range of electromagnetic wavelengths is shown in Fig. 4A. During the process of UV absorption, atoms or molecules experience transitions to higher energy levels as their outer electrons absorb photons. Consequently, the recorded optical absorption spectrum can be utilized for the determination of the energy band gap of the CeO_2_ NPs. The CeO_2_ NPs exhibit a discernible absorption peak at a wavelength of 321 nm in their UV–visible spectrum. The calculation of the optical band gap energy of CeO_2_ NPs was performed using Equation (1) [48]:

1 $$E_{\text{bg}=}\frac{1240}{\lambda }\left(eV\right)$$

In this context, λ represents the wavelength, while Ebg denotes the energy of the optical band gap. The determined optical band gap value of the synthesized CeO_2_ NPs was measured to be 3.86 eV, surpassing the reported value of 3.2 eV in previous studies for bulk CeO_2_ [49]. The observed increase in the optical band gap of CeO_2_ NPs can be attributed to the quantum size effect, which is in agreement with previous findings reported in the literature [50].

The XRD pattern (Fig. 4B) indicates that the synthesized NP_S_ possess a polycrystalline structure, specifically exhibiting the cubic fluorite CeO_2_ structure. Four distinct diffraction peaks are found at specific 2θ values: approximately 28.6°, 33.2°, 47.7°, and 56.6°. These peaks correspond to the crystallographic planes indexed as (111), (200), (220), and (311), respectively. The diffraction peaks seen exhibit a high level of concordance with the Joint Committee on Powder Diffraction Standard (JCPDS) No. 34–0394 [51]. No further peaks associated with impurities or other phases were observed in the XRD pattern, so confirming that the synthesized CeO_2_ NPs are composed only of crystalline CeO_2_ in a single phase. The DLS analysis (Fig. 4C) showed that the synthesized CeO_2_ NPs have a hydrodynamic diameter of around 105 nm. The FTIR spectrum of the synthesized CeO_2_ NPs was recorded over 400-4000 cm^−1^ and the results are presented in Fig. 4D. The band located at 3381.8 cm^−1^ can be attributed to the O–H vibration in absorbed water on the sample surface. The peak located at 1630 cm^−1^ can be related to the molecular H_2_O (H–O–H) bending frequency. The peak related to the CeO_2_ stretching vibration of CeO_2_ NPs can be seen around 440 cm^−1^. The purity of the synthesized CeO_2_ NPs can be concluded from the inexistence of the peaks at 2846.4 and 2918.7 cm^−1^, related to the CH_2_ vibrations of the applied source materials [48,[52], [53], [54]].

**Fig. 5:**
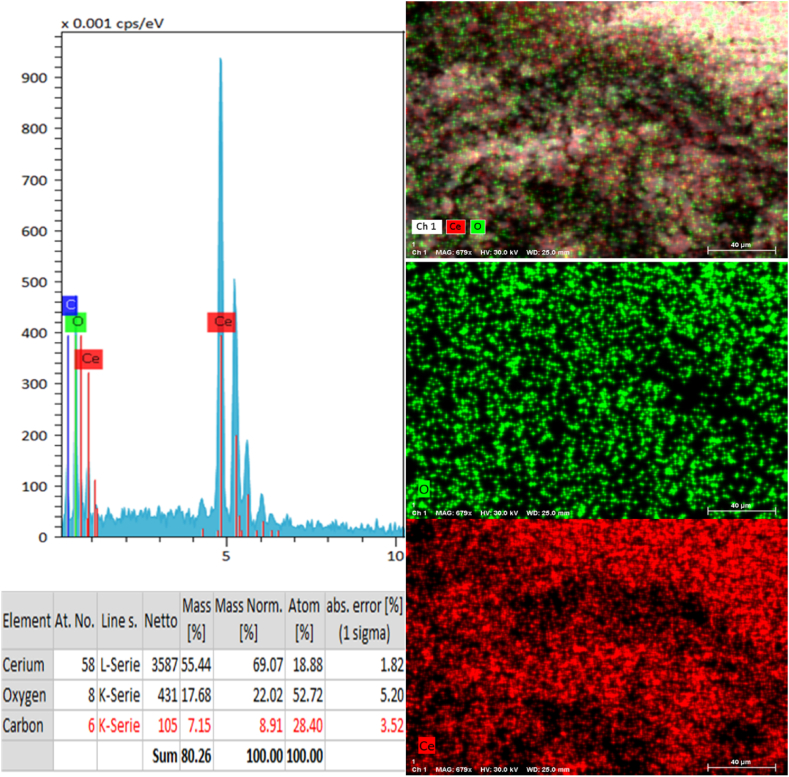
EDX spectra and Elemental mapping of the synthesized CeO_2_ NPs.

The analysis of the chemical composition of synthesized CeO_2_ NPs was conducted using EDX, as illustrated in Fig. 5. EDX analysis provided evidence supporting the absence of elements other than cerium (69.07 %) and oxygen (22.02 %) within the sample. Based on the analysis of the EDX spectrum, it can be concluded that the CeO_2_ NPs that were synthesized exhibited a high degree of purity, devoid of any surfactant or contaminant.

### 3.3 Antioxidant potential

**Fig. 6:**
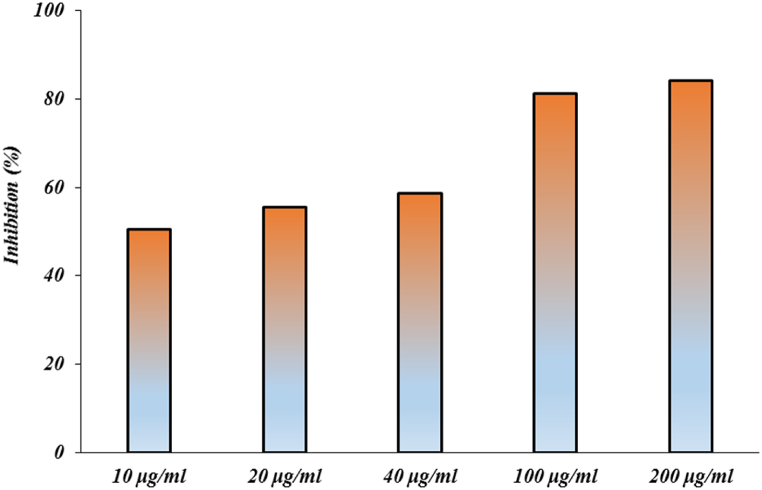
The DPPH radicals inhibition percentage of CeO2 NPs.

The ability of the synthesized CeO_2_ NPs to scavenge free radicals was evaluated using the DPPH antioxidant assay. Mutations in macromolecules including DNA, RNA, proteins, and lipids are caused by an imbalance between ROS generation and deactivation by the innate antioxidant system, which also speeds up cellular stress in cell signaling pathways. Moreover, in PD, oxidative stress contributes significantly to the death of dopamine-producing neurons. Interference with the neuron's redox potential disrupts several biological processes, which can lead to cell death. Key cellular components in the substantia nigra of people with PD have been shown to be damaged by oxidative and nitrative stress. Dopamine metabolism, mitochondrial failure, iron, neuroinflammatory cells, calcium, and ageing are all recognized as potential sources and processes for the formation of reactive oxygen species (ROS). DJ-1, PINK1, parkin, alpha-synuclein, and LRRK2, all of which are linked to PD, have complicated effects on mitochondrial function that increase ROS formation and vulnerability to oxidative stress. Oxidative stress also affects the ubiquitin-proteasome system and mitophagy, two cellular homeostatic mechanisms. In PD, neurodegeneration is likely caused by a feed-forward situation in which initial insults produce oxidative stress, which damages important cellular pathogenetic proteins, which in turn causes further ROS production, and so on. Fig. 6 represents the results of the antioxidant potential of the CeO_2_ NPs.

### 3.4 Induction of fibrillation in alpha-synuclein protein

**Fig. 7:**
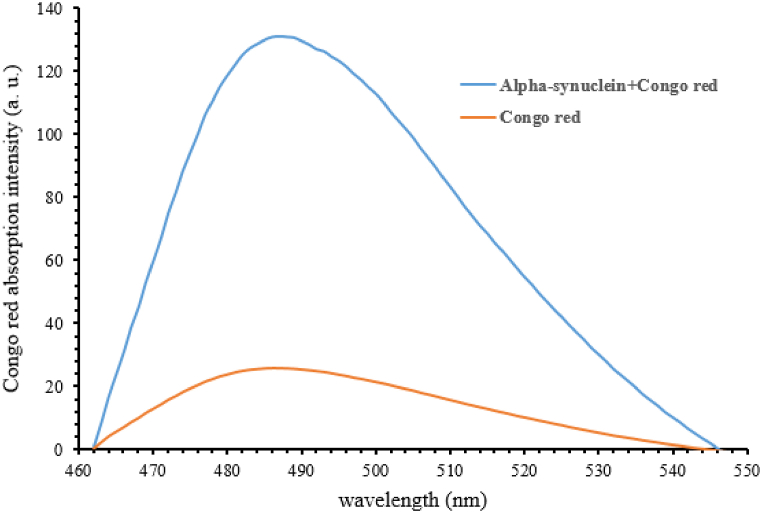
Absorption spectrum of Congo red α-synuclein heated (blue line) for 40 h at pH 7.2 and temperature 37° Celsius, Congo red as a control (Orange line). (For interpretation of the references to color in this figure legend, the reader is referred to the Web version of this article.)

In order to investigate the fibrillation of pure alpha-synuclein protein, samples were taken from the protein solution at different heating times under suitable conditions to induce the formation of fibrillar aggregates. The data obtained from the Congo red colorimetric test also showed that in a sample containing fibrillar accumulations of alpha-synuclein, in comparison with the color control sample, not only the light absorption intensity increased, but a transition to the red wavelength in its absorption spectrum is seen, which is one of the characteristics of Congo red binding, that is fibrillar structures (Fig. 7).

**Fig. 8:**
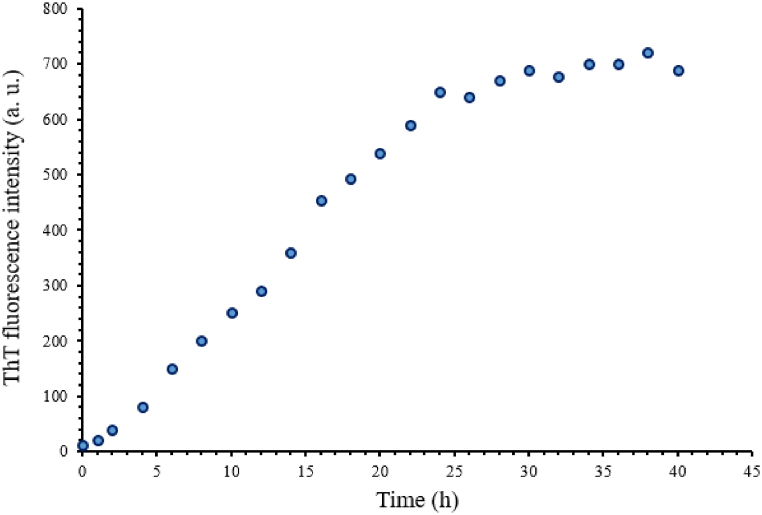
Induction of fibrillar aggregations in alpha-synuclein (0.5 mg/ml). Fluorescence emission spectrum of ThT after adding the synuclein sample which was heated for 40 h at pH 7.2 and temperature of 37° Celsius with a concentration of 0.5 mg/ml.

**Fig. 9:**
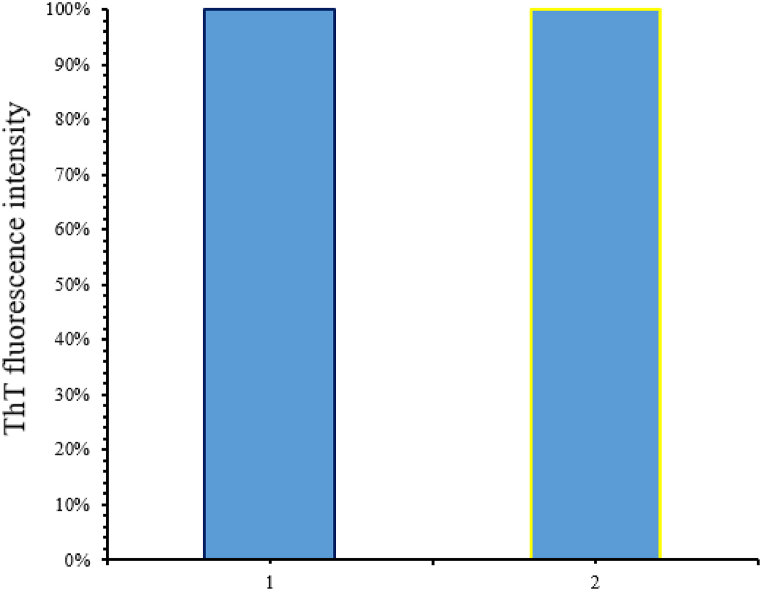
The fluorescence emission quenching effect of cerium oxide nanoparticles. After 40 h of alpha-synuclein incubation at 37 °C and pH 7.2, mature amyloid fibers were formed and its fluorescence emission was obtained in the absence of nanoparticles (left column). The fluorescence emission of amyloid fibers is also shown in the right column (number 2) immediately after adding 0.5 mg/ml nanoparticles.

Investigating the increase in fluorescence emission in the presence of ThT indicated an increase in absorption, and with increasing heating time, the intensity of fluorescence emission increased, which is one of the signs of the formation of fibrillar structures of beta sheets type (Fig. 8). So that increasing the intensity of thioflavin T fluorescence is one of the criteria for the presence of amyloid fibers. As Fig. 8 shows, this increase in emission intensity is attributed to the conversion of alpha-synuclein into amyloid fibers. After proving the conversion of alpha-synuclein to amyloid fibers, the effect of CeO_2_ NPs on inhibiting the formation of these aggregates was investigated. One of the controls that should be done is whether the reduction of fluorescence emission by nanoparticles is related to inhibition of amyloid accumulations by nanoparticles or is due to the quenching effect of the fluorescence spectrum in wavelengths between 460 and 550 nm by nanoparticles. To investigate the quenching effect, first, the fluorescence emission spectrum of mature amyloid fibers was read. Then the nanoparticles with a concentration of 0.5 mg/ml was applied on it and we immediately studied the intensity of its fluorescence emission. If there is a sharp decrease in emission, it is due to the quenching effect of the nanoparticles, not its inhibition. As seen in Fig. 9, adding nanoparticles to a solution containing mature amyloid and immediately observing its fluorescence spectrum does not show a decrease in emission intensity, therefore, the effect of the nanoparticle on the formation of amyloid fibers has an inhibitory effect and the decrease in fluorescence intensity is not due to its quenching effect.

### 3.5 Thioflavin-T (ThT) assay

**Fig. 10:**
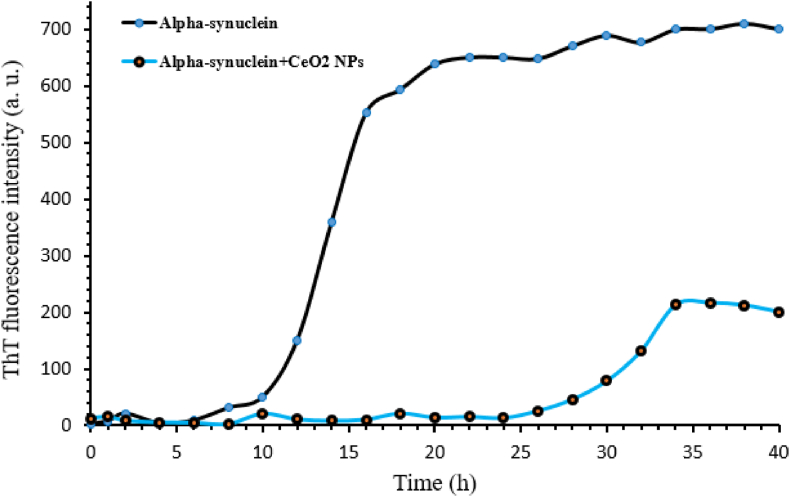
Kinetics of alpha-synuclein amyloid formation in the presence of CeO₂ NPs. The fluorescence emission spectrum of thioflavin T (ThT) of 1 mg of alpha-synuclein incubated at 37° Celsius and pH 7.2 in the presence (black circles) and absence (Blue circles) of nanoparticles at different times. (For interpretation of the references to color in this figure legend, the reader is referred to the Web version of this article.)

The fluorescence emission of 50 μM thioflavin T (ThT) solution in the presence of the protein sample incubated at 0–40 h was investigated, which is shown in Fig. 10. The process of changes in thioflavin T emission over time follows the sigmoid model, which includes three stages: the lag phase, the rapid growth stage, and the final equilibrium stage. The lag phase, which is called the nucleation phase, in this phase, the primary nuclei of the formation of amyloid fibers are obtained, and it is the limiting phase of the process, and after that, an exponential intensity of emission happens. The fluorescence emission of the protein sample was also investigated in the conditions of amyloid formation in the presence of 0.5 mg/ml of nanoparticles. As Fig. 10 shows, nanoparticles have a significant effect on the kinetics of amyloid fiber formation of alpha-synuclein protein, in such a way that both the fast growth phase and the final equilibrium phase show a decrease in terms of fluorescence intensity in the presence of nanoparticles compared to its absence. Of course, the increase in the lag phase and decrease in the growth phase in the presence of nanoparticles is the reason for the inhibitory effect of these nanoparticles.

Amyloid formation studies show that all proteins, regardless of whether they are pathogenic or non-pathogenic, are able to form amyloid fibers if placed in the right conditions. The fibers formed by these proteins are structurally and toxically no different from the fibers formed by the amyloid beta (Aβ) protein in Alzheimer's disease. Our studies showed that these nanoparticles are able to inhibit the formation of amyloid fibers in a concentration-dependent manner (with an effective concentration of 0.5 mg/ml). Also, these nanoparticles have an effect on the kinetics of aggregation formation and by prolonging the lag phase and reducing the growth phase, it has an inhibitory effect on this reaction.

### 3.6 Atomic force microscopy (AFM)

**Fig. 11:**
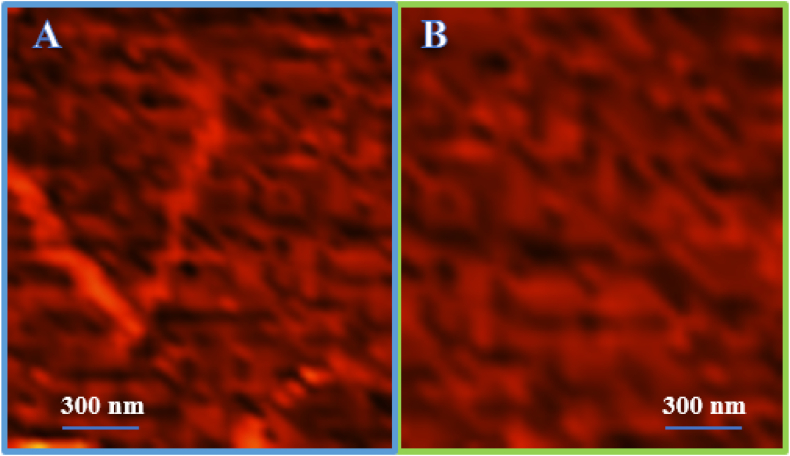
Atomic force Microscopy (AFM) images of α -Synuclein protein in the absence (A) and presence (B) of CeO_2_ NPs. The formation of amyloid fibrils is reduced in the presence of cerium oxide nanoparticles (B), (0.5 mg/ml of alpha-synuclein incubated at 37° Celsius and pH 7.2).

**Fig. 12:**
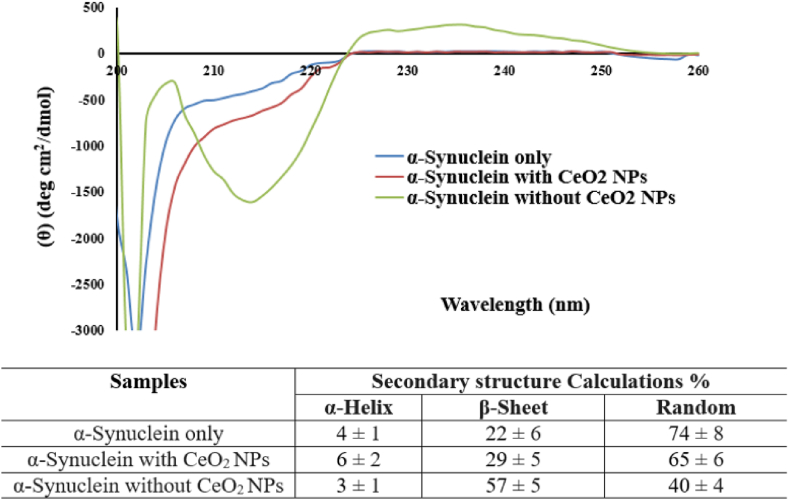
Far-UV CD spectra and circular dichroism evaluation of the secondary structure of α- Synuclein (0.5 mg/ml) in the presence and absence of CeO2 NPs (0.5 mg/ml).

Atomic force microscope imaging is a good complementary observation for the formation of alpha-synuclein protein amyloid fibrils. To further ensure the formation of amyloid structures, an atomic force microscope image was prepared from the prepared samples. This method is one of the most reliable tools used to prove the existence of amyloid aggregations. The obtained results clearly show fibrillar structures. As shown in Fig. 11, Incubation of alpha-synuclein protein in the absence (Fig. 11A) and presence (Fig. 11B) of cerium oxide nanoparticles showed a significant difference in the fibrillation process. As shown in Fig. 11B, the formation of fibrils of alpha-synuclein protein in the presence of cerium oxide nanoparticles is strongly reduced, which confirms the results of CD (Fig. 12) and other techniques.

### 3.7 Circular dichroism (CD) spectroscopy

Fig. 12 shows the CD spectrum of alpha-synuclein protein in the presence and absence of incubation with CeO_2_ NPs in the far ultraviolet range (190–260 nm). The appearance of the broad and deep negative peak in the absence of CeO_2_ NPs in the CD spectrum around 215–225 nm, is considered as the indicator of the formation of open and wide intermolecular beta type structures (fibrillar shape). As the results of deconvolution of CD spectrum show (inserted table), in the presence of cerium oxide nanoparticles, the formation of beta sheet structures is greatly reduced compared to its absence, and the structure is close to the natural structure of alpha-synuclein protein.

### 3.8 Quantum calculations of CeO_2_ and levodopa

Quantum calculations are powerful tool to investigate molecular structures and optimization of the equilibrium geometry [55]. In order to obtain the most stable configurations and their energies, DFT-D was applied by DMol3 module in Materials Studio 2017. The optimization energies of CeO₂ and Levodopa were achieved −34853.893 and −699.987 Ha, respectively. Depending on negative data based on DFT-D, all structures were obtained stable, resulting that all stable compounds are represented in Fig. 1 e and f.

**Fig. 13:**
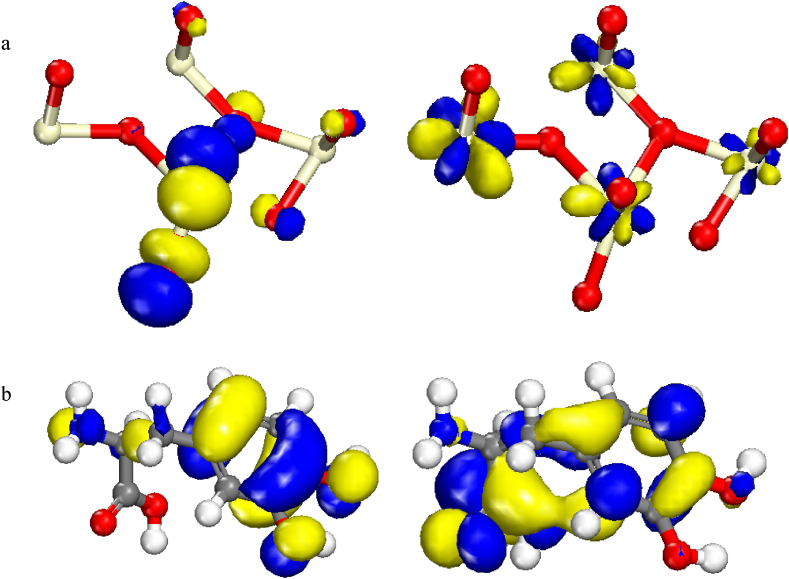
HOMO and LUMO orbitals of (a); CeO₂ and (b); Levodopa based on DFT-D in Materials Studio 2017.

HOMO orbitals of CeO_2_ were distributed on some O and Ce, meanwhile, LUMO orbitals of CeO_2_ were located on Ce atoms as represented in Fig. 13a. HOMO orbitals of Levodopa were located on benzene ring, hydroxyl atoms and N atom. In addition, LUMO orbitals of Levodopa were located on overall molecule exception hydroxyl atoms as represented in Fig. 13b. The obtained HOMO-LUMO energy gaps of CeO_2_ and Levodopa are 0.145 and 4.012 eV, respectively. According to the values of gaps, the reactivity ranking of CeO_2_ is more than that of Levodopa, indicating that CeO_2_ is more reactive than Levodopa compared with receptor.

### 3.9 Docking analysis of the molecular structures

**Fig. 14:**
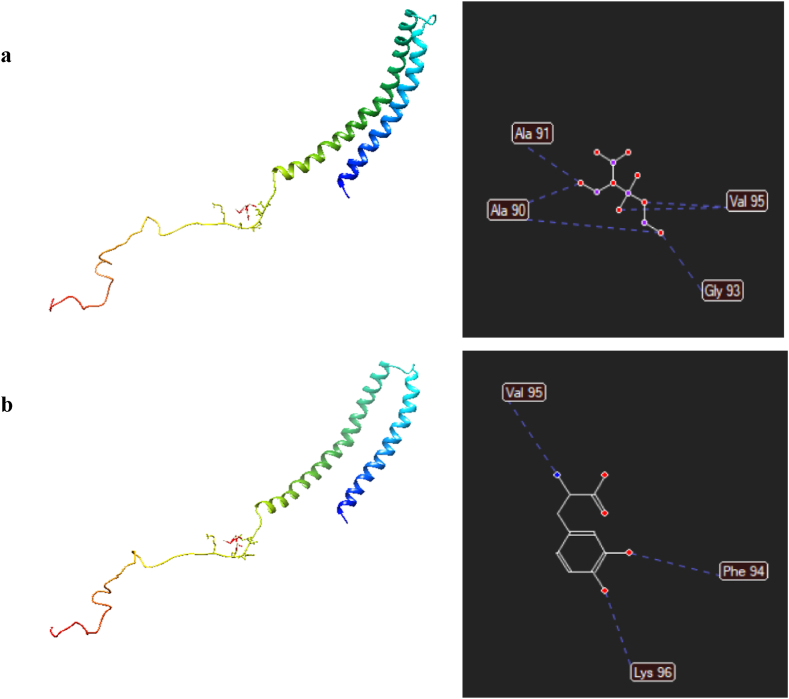
Plot (3D and 2D) of the principal component analysis of hydrogen interactions of (a); CeO₂ and (b); Levodopa with α-synuclein (1XQ8).

Inhibition of α-synuclein protein aggregation is still a challenge to treat Parkinson's disease. Levodopa is widely used in the clinic on the progression of the disease and prevented aggregation [56]. Nowadays, molecular dynamic and molecular docking have been become an essential part of the drug discovery process to reduce time and costs [57,58]. For this aim, CeO_2_-based (111), as the highest peak in XRD, was prepared and applied without cell unit. The minimization energy values of interaction between CeO_2_ and Levodopa with α-synuclein at the most stable structures were calculated −59.313 and −39.656 kcal mol^−1^, respectively (Fig. 14a and b). CeO₂ was formed hydrogen interactions with Ala90 (−9.664 kcal mol^−1^), Ala91 (−6.587 kcal mol^−1^), Gly93 (−5.235 kcal mol^−1^) and Val95 (−11.496 kcal mol^−1^) along with electrostatic interactions Phen94 (−13.225 kcal mol^−1^) and Thr92 (−0.323 kcal mol^−1^). Meanwhile, Levodopa had hydrogen bonds with parallel orientation containing of Lys96 (−15.171 kcal mol^−1^), Val (−1.062 kcal mol^−1^) and Phe94 (−20.894 kcal mol^−1^). The minimum free energy binding of both two compounds were negative, resulting interaction between compounds and protein [59]. Aa a result, these compounds inhibited α-synuclein protein aggregation. In addition, that CeO_2_ strongly binds with receptor by relative binding energy as compared with Levodopa [60].

**Fig. 15:**
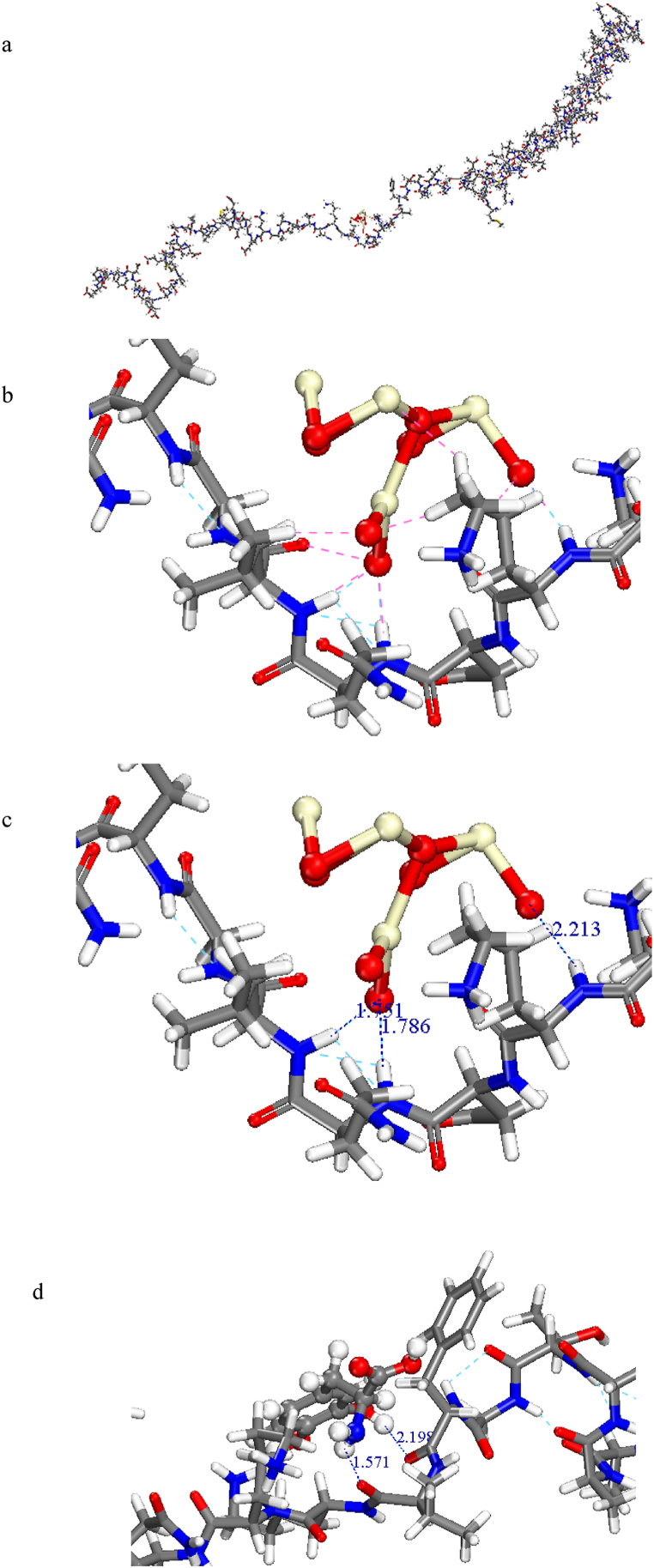
The equilibrium distances and hydrogen bonds between (a–c); CeO₂ or (d); Levodopa and *α*-synuclein (1XQ8).

Furthermore, the interaction between CeO_2_ and receptor are represented in Fig. 15a. Indeed, the existence of hydrogen bonding between O atoms of CeO_2_ and N–H of adjacent acid amines and the equilibrium distances were confirmed by 1.751 Å (Leu100), 1.786 Å (Gln99) and 2.213 Å (Lys97) as shown in Fig. 15b and c. Meanwhile, the equilibrium distances between the nearest atoms of N–H in Levodopa and acid amines of the α-synuclein were obtained 2.198 Å (Phe94) and 1.571 Å (Lys96), resulting the hydrogen bonds (Fig. 15d).

Parkinson's and Alzheimer's are among the neurological diseases, the origin and cause of which is unknown. Many hypotheses have been proposed in the last few decades about the causes of these diseases, but none of them were successful, because no specific treatment solution has been provided. One of the strongest and most important hypotheses in these diseases is the hypothesis of the accumulation of proteins that do not respond to drugs (undruggable protein), which is known as the amyloid hypothesis. These protein aggregates usually have common features, including the creation of intertwined structures called amyloid structures. Inhibiting the formation of these amyloid structures has been of great interest to researchers. One of the most important inhibitors in this regard is the use of nanomaterials.

Anupam Maity et al. used naringenin-functionalized Gold Nanoparticles to inhibit the fibrillation process of alpha-synuclein proteins. The binding affinity of nanoparticles to alpha-synuclein protein was in the micromolar range, and structural studies (Circular Dichroism study) showed that it prevented the formation of fibrillation process and beta-sheet structures. Also, nerve cell survival studies in the presence of naringenin revealed that naringenin does not have negative effects on the survival of nerve cells [61].

In another study, Lisni P. Sunny et al. investigated the interaction of tryptophan-cardanol nanoparticles with the monomeric and fibrillary structures of alpha-synuclein proteins. The inhibition of the formation of amyloid structures in this study was concentration-dependent, and at the optimal concentration of 5 μM of these nanoparticles, inhibitory properties were observed. In addition to inhibiting the formation of amyloid structures, these nanoparticles showed the decomposition of amyloid structures, after 48 h of incubation of amyloid structures with these nanoparticles. The effects of SH-SY5Y cell line survival in the presence of these nanoparticles were investigated and it was shown that the nanoparticles had no cytotoxic properties [62].

The inhibitory effects of various nanomaterials on the process of fibrillation formation in neurodegenerative diseases have been investigated from different point of view (interaction affinity, structural changes, fibrillation process, and etc.). The study of the interaction of cerium oxide nanoparticles on the fibrillation process of alpha-synuclein protein from the theoretical, experimental point of view and the optimization of the oxidative stress conditions (antioxidant properties) of Parkinson's disease has been done in this study for the first time. Compared to previous studies, cerium oxide nanoparticles showed a relatively good inhibitory effect on alpha-synuclein fibrillation inhibition process. Quantitative studies (kinetic study, biophysical study and so on.) with more details are suggested to determine the interaction of cerium oxide with alpha-synuclein protein for a more precise design of drugs with fibrillation inhibition properties.

## 4 Conclusion

A significant number of human diseases, including diseases that destroy the nervous system, It depends on the formation of protein plaques known as amyloid fibrils, and for this reason, they are also called amyloid diseases. In unknown conditions, alpha-synuclein structure changes and makes it prone to entering the fibrillation process and creating beta-sheet structures (amyloid diseases condition). Studies have shown that some materials, including polymeric nanoparticles, metal nanoparticles and etc., are able to inhibit the accumulation and cellular toxicity of alpha-synuclein protein. Free radicals are created during oxidative stress in cells, which are considered one of the destructive factors in neurodegenerative diseases. The antioxidant activity of nanoparticles is directly related to their neuroprotective effects. In this study, cerium oxide nanoparticles were synthesized and characterized by using different technique (structural and functional properties such as morphology, size, size distribution, elemental analysis, purity, antioxidant and antifibrillar activities). The results of microscopic studies (TEM & SEM) showed a uniform morphology and monodispersed (around 100 nm) nanoparticle distributions, which confirmed the DLS data (105 nm). Chemical composition (EDX), purity (FTIR) and optical absorption spectra (UV–Vis) of the synthesized CeO2 NPs were characterized, and the results showed that cerium oxide nanoparticles are pure and have polycrystalline morphology. The results of structural and microscopic investigations of nanoparticles showed that the nanoparticles are suitable for biomedical applications. Synthesized cerium oxide nanoparticles showed strong antioxidant (DPPH assay) and antifibrillar properties (CD, AFM and fluorescence studies). The interaction of cerium oxide nanoparticles with alpha-synuclein proteins from the mechanistic point of view using theoretical information (Quantum calculations & Docking) showed a specific and appropriate interactions manner. Kinetic studies of the interaction of CeO_2_ NPs with alpha-synuclein protein are suggested for further studies in order to determine the details of this interaction. The results of antioxidant and antifibrillar studies of cerium oxide nanoparticles can be promising to optimize the conditions of Parkinson's disease caused by the accumulation of alpha-synuclein protein.

## Funding

Not applicable.

## Data availability statement

Data will be made available on request.

## CRediT authorship contribution statement

**Xiaomei Yao:** Writing – original draft, Investigation. **Yichao Guan:** Investigation, Formal analysis, Data curation. **Jianli Wang:** Investigation, Formal analysis, Data curation. **Dong Wang:** Writing – original draft, Investigation.

## Declaration of competing interest

The authors declare that they have no known competing financial interests or personal relationships that could have appeared to influence the work reported in this paper.
